# Data of cost-optimal solutions and retrofit design methods for school renovation in a warm climate

**DOI:** 10.1016/j.dib.2016.11.004

**Published:** 2016-11-05

**Authors:** Ilaria Zacà, Giuliano Tornese, Cristina Baglivo, Paolo Maria Congedo, Delia D’Agostino

**Affiliations:** a Department of Engineering for Innovation, University of Salento, 73100 Lecce, Italy; b Energy efficiency and Renewables Unit, Institute for Energy and Transport (IET), Joint Research Centre (JRC) - European Commission, Ispra, VA, Italy

**Keywords:** School, Refurbishment, Zero energy buildings (ZEBs), EPBD, Recast, Cost-optimal

## Abstract

"Efficient Solutions and Cost-Optimal Analysis for Existing School Buildings" (Paolo Maria Congedo, Delia D’Agostino, Cristina Baglivo, Giuliano Tornese, Ilaria Zacà) [1] is the paper that refers to this article. It reports the data related to the establishment of several variants of energy efficient retrofit measures selected for two existing school buildings located in the Mediterranean area. In compliance with the cost-optimal analysis described in the Energy Performance of Buildings Directive and its guidelines (EU, Directive, EU 244,) [2], [3], these data are useful for the integration of renewable energy sources and high performance technical systems for school renovation. The data of cost-efficient high performance solutions are provided in tables that are explained within the following sections.

The data focus on the describe school refurbishment sector to which European policies and investments are directed. A methodological approach already used in previous studies about new buildings is followed (Baglivo Cristina, Congedo Paolo Maria, D׳Agostino Delia, Zacà Ilaria, 2015; IlariaZacà, Delia D’Agostino, Paolo Maria Congedo, Cristina Baglivo; Baglivo Cristina, Congedo Paolo Maria, D’Agostino Delia, Zacà Ilaria, 2015; Ilaria Zacà, Delia D’Agostino, Paolo Maria Congedo, Cristina Baglivo, 2015; Paolo Maria Congedo, Cristina Baglivo, IlariaZacà, Delia D’Agostino,2015) [4], [5], [6], [7], [8]. The files give the cost-optimal solutions for a kindergarten (REF1) and a nursery (REF2) school located in Sanarica and Squinzano (province of Lecce Southern Italy). The two reference buildings differ for construction period, materials and systems.

The eleven tables provided contain data about the localization of the buildings, geometrical features and thermal properties of the envelope, as well as the energy efficiency measures related to walls, windows, heating, cooling, dhw and renewables. Output values of energy consumption, gas emission and costs are given for a financial and a macro-economic analysis.

This data article provides 288 and 96 combinations for REF1 and REF2, respectively. The output values are obtained using the software ProCasaClima 2015v.2.0.

**Specifications Table**

**Table:** 

Subject area	*Civil engineering*
More specific subject area	*High energy efficiency solutions for buildings with school use*
Type of data	*Tables*
How data was acquired	*Technical datasheets, Puglia Region price list, market surveys, ISTAT surveys, software ProCasaClima 2015v.2.0*
Data format	*.xls*
Experimental factors	*No pretreatment of samples was performed*
Experimental features	*Cost-optimal solutions have been derived for several combinations of energy efficiency technical variants, applied to two reference buildings, with school use, located in Southern Italy.*
Data source location	*Lecce – Italy, Mediterranean climate*
Data accessibility	*Data is provided in Supplementary materials directly with this article*

**Value of the data**

• Identification of efficient measures following the cost-optimal methodology to find the best solutions for nearly zero energy buildings (nZEBs).
• Definition of several combinations of energy efficiency measures for refurbishment in the school sector.
• Evaluation of cost-optimal energy measures in terms of primary energy consumptions and global costs for two existing school buildings located in the Mediterranean climate.

## 1 Data

Input and output data are provided in the eleven tables containing, in particular, geometrical features, thermal properties of external walls and windows, characteristics of technical systems, as well as primary energy consumptions, CO_2_ gas emissions and global costs for two reference buildings located in province of Lecce (national climatic Zone C, Southern Italy).

## 2 Experimental design, materials and methods

Energy efficiency measures have been identified for two existing reference buildings by the implementation of a methodology that includes several steps, starting from the characterization of the reference buildings, the definition of several appropriate efficiency measures, and the evaluation of the energy performance and the global costs.

The data are derived for two school buildings located in the Mediterranean climate that is characterized by non-extreme winters and high aridity summers, with rainfall mainly occurring in autumn and winter [9].

The input data about the geometrical features (e.g. perimeter, area, volume, shape factor) are obtained from existing documents about the building design. The technical systems data have been obtained from available technical specifications considering the age of the structures and their implementation.

The first spreadsheet attached (.xls) in attachment includes the tables in sheets S1.A-B-C-D. They give the description about geometrical features, the construction element properties, thermal properties of external walls and windows of the two existing reference buildings described in details in [1].

The description of the variants measures is reported in the second spreadsheet attached (.xls). In more details, the worksheet S2.A contains the tables in which the variants of the first reference building (REF1) are listed, while the sheet S2.B refers to the second one (REF2) These measures are related to the different types of insulation and windows. In particular, the thermal properties and thickness of insulating materials window glazing are described. Moreover, a detailed description of the materials used for the renovation of the roof, the internal slab and the floor are given. The third sheet in the same file, S2.C, contains the table related to the technical system variants both for REF1 and REF2. The variants for generation, emission, ventilation and renewable energy sources systems are given for each reference building.

The tables in worksheets S3.A-B-C-D, in the third spreadsheet (.xls) in attachment, give all the combinations of variants related to the adopted measures. In particular, tables in worksheets S3.A-B give the combinations and the output values of the first existing school building, while S3.C-D include the data for the second one, both for a financial and a macroeconomic analysis. For each combination the following outputs have been obtained:

• CO_2_ gas emission (kgCO_2_/m^2^y);
• Primary energy consumption (kWh/m^2^y);
• Global costs (€/m^2^);
• Energy classification (according to the standard CasaClima);
• CO_2_ emission and primary energy reduction in comparison to the reference buildings.

The study contributes to define the strategies to reach cost-optimal high performance building retrofit in a warm climate according to national requirements and the European Directive [2], [3]. This application, with other studies [4], [5], [6], [7], [8], provides a complete view of the possible actions on buildings with different uses, in order to reach the ZEB level.

## Author contributions

All authors participated in preparing the research during its different phases, such as establishing research design, methods and analysis. The authors discussed and finalized the results together and prepared the manuscript according to the progress of the research.

## Footnotes

Appendix A Supplementary data associated with this article can be found in the online version at 10.1016/j.dib.2016.11.004.
